# Maine Veterinary Medical Association

**Published:** 1896-09

**Authors:** W. L. West

**Affiliations:** Secretary


### MAINE VETERINARY MEDICAL ASSOCIATION.

THE semi-annual meeting was called to order by President Russell, and upon roll-call the following members answered to their names: Drs. Russell, Choate, Huntington, Freeman, Salley, Purcell, Stevens, and West, with Cain, of Norway, a visitor. Reading of minutes of preceding meeting postponed.

Dr. Purcell reported for the committee appointed to confer with the State Cattle Commission, and said the members were received and treated throughout with great courtesy, and they agreed to vouch for no more non-graduates as capable of testing cattle, and to restrict themselves to those already vouched for. Dr. Purcell exhibited a letter from Hon. F. O. Beale embodying the gist of the report. On motion the report was accepted.

On motion of Dr. West a memorial was forwarded to United States Senators Hale and Frye, asking them to do all in their power to defeat Senate Bill 1552, otherwise known as the antivivisection bill, which will come before the Senate at its next session. This question excited considerable discussion from Drs. Huntington, Purcell, Choate, and West, but was finally carried, and the Secretary was instructed to send the memorial.

The Secretary read a letter from G. H. Bailey tenderlng his resignation from the Association, and it was unanimously voted not to accept it.

Dr. Purcell called Dr. Choate to account for an alleged breach of ethics in trying to obtain practice by offering his services for lower prices than the others.

On motion of Dr. Purcell, the Association condemned any member who publicly endeavors to obtain practice by offering his services below the prices charged by any other member.

On motion of Dr. Salley, the Association voted to reconsider the schedule of prices, so that in future no member shall test cattle for less than one dollar per head.

Drs. Russell and Choate thought we should express our sentiments to the effect that the tuberculin test of cattle as applied by non-professional men is misleading and very liable to be misunderstood by the public.

On motion of Dr. Huntington the following resolutions were unanimously adopted:

*Resolved*, That the Maine Veterinary Medical Association respectfully protest that the testing of cattle for export to Massachusetts or for furnishing milk to our own citizens is a matter of such vital importance that it should only be done by competent and well-qualified men, and so far as it is being done by men of no professional training the results may be misleading and of no value.

*Resolved*, That if this important test cannot be made by professional men, there is no valid reason for its being made at all, as it entails an unwarranted expense upon the owner or shipper.

The Secretary was instructed to forward a copy of the above resolutions to the Maine and Massachusetts Secretaries of the Cattle Commissions, the President of the Maine Board of Health, and to all State newspapers.

On motion, it was voted that the following bill, presented to the Maine Legislature in 1893–94, be again presented at the coming meeting:

Be it enacted by the Senate and House of Representatives in Legislature assembled as follows:

Section 1. No person shall advertise or hold himself out as a veterinarian or veterinary surgeon unless he is a graduate of a veterinary college or school.

Sect. 2. Any person violating the provisions of the foregoing section shall be punished by a fine of not less than five dollars nor more than fifteen dollars, or by imprisonment not more than three months or both.

W. L. West, V.S.,
Secretary.

